# A Model for the Evolution of Extremely Fragmented Macronuclei in Ciliates

**DOI:** 10.1371/journal.pone.0064997

**Published:** 2013-05-21

**Authors:** David W. Morgens, Kristen M. Lindbergh, Marie Adachi, Ami Radunskaya, Andre R. O. Cavalcanti

**Affiliations:** 1 Department of Biology, Pomona College, Claremont, California, United States of America; 2 Department of Mathematics, Pomona College, Claremont, California, United States of America; Laboratoire de Biologie du Développement de Villefranche-sur-Mer, France

## Abstract

While all ciliates possess nuclear dimorphism, several ciliates – like those in the classes Phyllopharyngea, Spirotrichea, and Armophorea – have an extreme macronuclear organization. Their extensively fragmented macronuclei contain upwards of 20,000 chromosomes, each with upwards of thousands of copies. These features have evolved independently on multiple occasions throughout ciliate evolutionary history, and currently no models explain these structures in an evolutionary context. In this paper, we propose that competition between two forces – the limitation and avoidance of chromosomal imbalances as a ciliate undergoes successive asexual divisions, and the costs of replicating massive genomes – is sufficient to explain this particular nuclear structure. We present a simulation of ciliate cell evolution under control of these forces, allowing certain features of the population to change over time. Over a wide range of parameters, we observe the repeated emergence of this unusual genomic organization found in nature. Although much remains to be understood about the evolution of macronuclear genome organization, our results show that the proposed model is a plausible explanation for the emergence of these extremely fragmented, highly polyploid genomes.

## Introduction

Ciliates are large unicellular eukaryotes characterized by nuclear duality and the presence of cilia. Each ciliate cell possesses two different types of nuclei: a transcriptionally silent, germline nucleus (micronucleus – MIC) used to transfer DNA during sexual conjugation and a transcriptionally active, somatic nucleus (macronucleus – MAC) used for vegetative growth [1], [2]. The organization of the DNA in the MIC is typical for eukaryotes: diploid nuclei comprised of several large chromosomes each containing hundreds or thousands of genes and significant amounts of non-coding material.

The MAC of ciliate cells is generated from the MIC in a process that involves DNA elimination, fragmentation, and amplification of chromosome numbers. The final organization of DNA in the MAC varies widely between ciliate species. Among the species for which the macronuclear genome has been sequenced, *Tetrahymena thermophila*'s MAC contains 225 chromosomes each present in 45 copies [3], [4]; *Ichthyophthirius* contains 71 chromosomes with a ploidy of ∼12,000 copies [5]; *Paramecium tetraurelia* contains about 200 chromosomes with a ploidy of ∼800 copies [6], [7]; *Oxytricha trifalax*, contains ∼15,600 chromosomes with a variable ploidy of ∼1,900 copies [8]. The extreme fragmentation seen in *Oxytricha* is also present in several other spirotrich species. The level of DNA elimination in the conversion of the MIC into the MAC is also highly variable among ciliate species. In the extreme case of Spirotrichs, only 2% of the total micronuclear genome sequence is present in the MAC. In addition to the better-characterized Spirotrichs, similar nuclear organization seems to have evolved independently in several other ciliate classes, such as Phyllopharyngea and Armophorea [9]. Even though this peculiar genomic organization has been recognized for some time, no evolutionary model has been advanced to account for how it could have emerged.

In this paper, we present a model for how the peculiar genomic organization of spirotrichs – extremely high ploidy, greatly reduced genome complexity and thousands of single-gene chromosomes – might have evolved. In particular, we will show that the unique way in which these nuclei divide can lead to selective pressures to increase ploidy and chromosome fragmentation and thus the generation of macronuclei with high ploidy and extensively fragmented chromosomes.

### The ciliate cell cycle

During vegetative growth, characterized by a lack of sexual conjugation, the MAC generates all transcripts needed for protein synthesis. Experiments involving amicronucleated ciliates have shown that some ciliates like *Tetrahymena* can survive and continue to divide asexually without a MIC, while others cannot [1]. During each asexual division, the MIC replicates via mitosis generating daughter cells with identical MICs. The MAC, on the other hand, divides via amitosis – a poorly understood, stochastic process that divides the genetic material of the MAC into two roughly equivalent nuclei without the aid of centromeres or metaphase plates [1], [10].

During sexual conjugation, the MICs of the two conjugating cells undergo meiosis, and one of the resulting haploid nuclei is exchanged between the partners. The transferred haploid copy fuses with one parent copy to form the zygotic MIC. Following conjugation, the MAC deteriorates, and the zygotic MIC generates a new MAC [1]. The differences between the DNA organization in the MAC and the MIC highlight the complexity of the process by which a new MAC is formed. This process involves elimination of non-coding DNA sequences, chromosome fragmentation, and DNA amplification to generate the unique features of the MAC, but the mechanism is still poorly understood. Recently, a consensus seems to be emerging that the old MAC contributes to the formation of the new MAC through epigenetic processes [11], [12].

Although the broad strokes of the ciliate cell cycle are reasonably well understood, few studies concentrated on determining the dynamics of growth of these cells. One notable exception is a systematic study of the life cycle of the Spirotrich *Sterkiella histriomuscorum* [13], [14]. This study provides the most detailed information about the spirotrichous ciliate life cycle available, so we rely on these results for the design and parameterization of our simulations.

In part due to the creation of the MAC, conjugation is a time consuming process: in *S. histriomuscorum*, the first vegetative cell division occurs 2.5 days after the start of conjugation, while a full asexual cycle can take as little as 5 hours [14]. Following this first vegetative division, the cells divide at a decreasing rate for the rest of their lifespan. During this period, the daughter cells are only fertile - able to conjugate sexually - between 25 and 80 vegetative generations [14].

### Amitosis and Chromosome Imbalances

Amitosis entails the duplication of the MAC chromosomes followed by the random division of the chromosomes to daughter nuclei. Given its stochastic nature, amitosis is only viable due to the genomic structure of the macronucleus. In a diploid nucleus, such as the MIC, the uneven division of chromosomes would likely result in severe chromosome imbalances – with both daughter cells missing some chromosomes and containing extra copies of others. The severity and the probability of imbalances depend on the ploidy, number of chromosomes, and existing imbalance of the parent nucleus. Kimura [15] developed an analytical formula for the probability that a cell will not completely lose any chromosome after **G** generations of amitotic division (Eq 1):
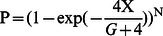
(1)where **N** is the number of different chromosome types, **X** is the number of copies of each distinct chromosome type, and **G** is the number of amitotic divisions since the last sexual conjugation. The simple conclusion behind this formula is that the probability of a chromosome being eliminated increases with the number of amitotic divisions and the number of distinct chromosome types – referred to as chromosome number – and decreases with the number of copies of each chromosome – referred to as copy number [15].

Kimura's model assumes uniform copy numbers for all MAC chromosomes. In absence of this assumption, analytical solutions become impractical. A more recent study used computational simulations to show that the same major conclusions hold for nuclei with different copy numbers for each chromosome [11]. In these cases, the copy number of the chromosome with the smallest number of copies determines the probability of chromosome loss.

Although it is not clear if chromosomal loss due to amitosis causes senescence in ciliate lineages, and indeed some authors have argued otherwise [16], the above studies suggest that, in absence of a regulatory mechanism to control copy number, multiple rounds of amitosis inevitably lead to the loss of chromosomes and consequent cell death. This effect would be greater in organisms with low copy numbers, as unsuccessful divisions would occur too frequently to sustain adequate growth. It should be noted that even without the complete elimination of a chromosome, imbalances should lead to dose dependent fitness effects [17], [18]. This is consistent with observations that vegetative ciliate cultures take progressively longer and longer to duplicate [14]. In fact, a recent mutation accumulation study concluded that fitness costs associated with chromosome copy number variations are responsible for the rapid collapse of vegetative *Tetrahymena* populations [18].

Following sexual conjugation in ciliates, the post-zygotic MIC gives rise to a new balanced MAC, thus the imbalances accumulated during vegetative growth by amitosis are reset by sexual conjugation. Yet sexual conjugation is a time consuming process that does not lead to population growth. This creates an impasse for ciliate cells: a lineage that grows mostly asexually would potentially have a much faster rate of population growth than a lineage that undergoes frequent conjugation, yet the cells in the primarily asexual lineage will accumulate more chromosome imbalances with a consequent decrease in fitness. Our model proposes that the extremely fragmented MAC of some ciliates evolved to avoid this lose-lose situation.

### The Model

We posit that early ciliate cells could decrease the level of chromosome imbalances by increasing the ploidy of the macronucleus. An increase in the copy number of chromosomes would minimize the effects of stochastic sampling (Eq. 1). Yet extreme polyploidy, and the requisite increase in DNA content, has its own drawbacks.

Previous works demonstrated a positive linear relationship between mitotic cycle time and cell size as well as between cell size and quantity of DNA [19], [20]. Later papers argued that the quantity of DNA within a cell actually determines both the cell size and the mitotic cycle time [21], though there is some data against this correlation [22]. Although other factors influence cell cycle time, an established, strong positive correlation exists between DNA content and division time. This applies specifically in ciliates with respect to amitosis as well [23], [24], [25]. An increase in ploidy thus leads to longer amitotic division times and slower cell growth, which decreases fitness.

We postulate here that the large amounts of non-coding DNA elimination during the formation of the MAC might have evolved as a mechanism to avoid the linear growth of DNA content associated with higher ploidy [1]. DNA elimination can lead to higher fragmentation and a larger number of different chromosome types [26]. This increase in the number of unique chromosomes results in more imbalances and thus increased pressure to augment copy number again (Eq. 1), creating a positive feedback loop of ploidy amplification necessitating DNA elimination and consequent fragmentation, in turn necessitating more ploidy amplification.

Our model proposes that the interplay between these two selective pressures – chromosome imbalances and DNA content – is the driving evolutionary force behind the peculiar nuclear organization of some ciliates. Given the relative lack of quantitative data on ciliate life cycle and evolutionary history, the goal of this paper is to use a computer simulation of ciliate populations to establish the plausibility of our model. We will show that for a large range of parameter values these two selective forces will lead to the emergence of extensively fragmented, high-ploidy macronuclei.

## Materials and Methods

**Figure 1:**
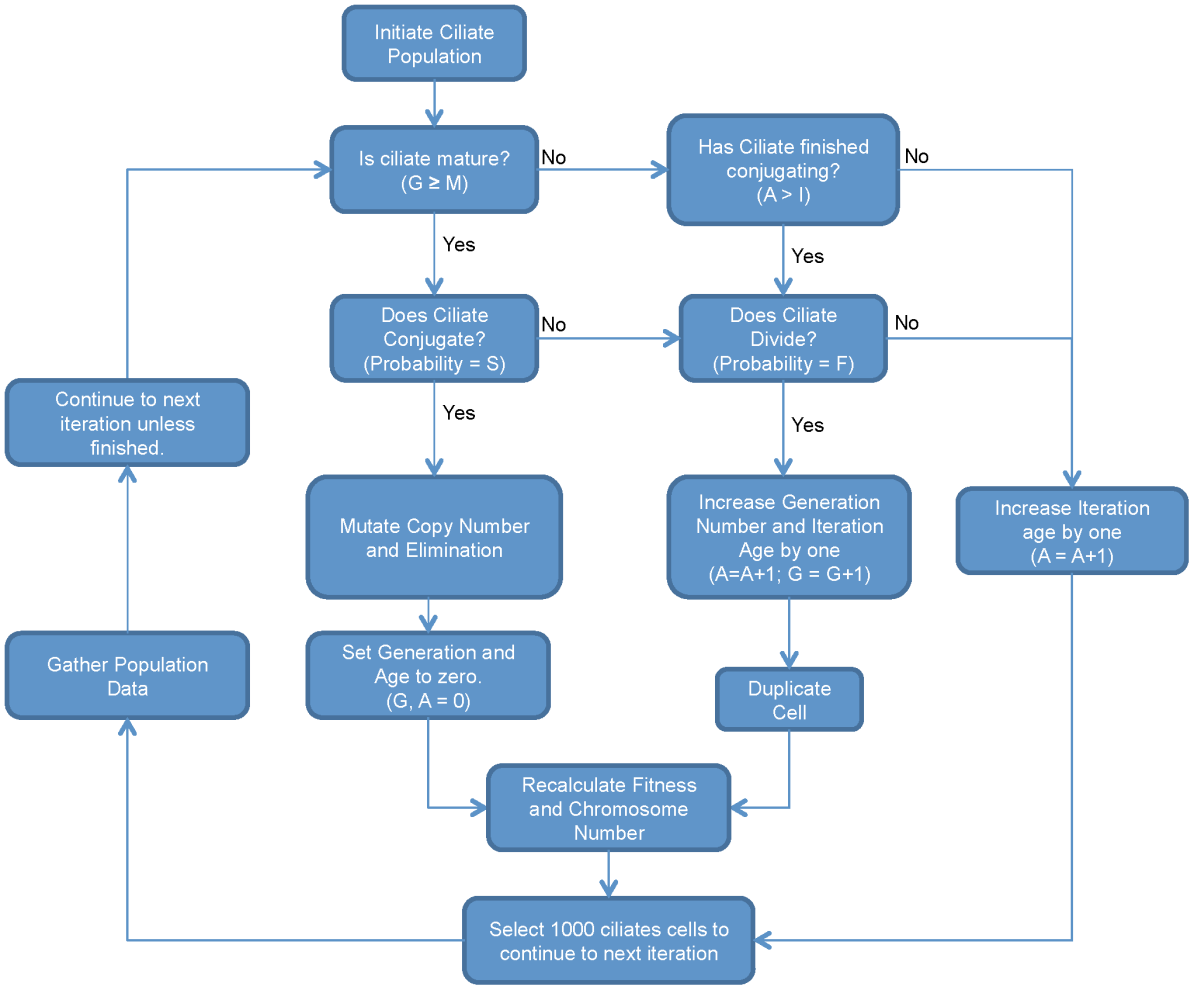
Flowchart of simulation. Cycles continue for 100,000 iterations.

We performed multiple computer simulations of the evolution of ciliate populations with varying parameters. Each trial contained 1,000 ciliate cells. These populations undergo 100,000 successive iterations under the assumptions of our model. At every iteration, each ciliate can conjugate sexually, divide asexually, survive to the next iteration, or be eliminated from the population (Figure 1).

Some parameters remain constant for all cells in the simulation, namely the probability of conjugation, **S**, and the fitness functions. In addition to these population wide parameters, each cell in the population has several individual attributes: copy number, **X**, amount of DNA eliminated, **E**, chromosome number, **N**, and fitness, **F** (Table 1, 2).

**Table 1:** Definition, default values and ranges for parameters used in the simulations.

Abbreviation	Description	Default Value	Value Range
X_0_	Initial Copy Number	10	10–50
N_0_	Initial Chromosome Number	50	35–75
Sd_copy_	Standard Deviation for Copy Number	5	5–50
Sd_elim_	Standard Deviation for Elimination coefficient	.01	0.01–0.05
I	Required Iteration Age to Divide	2	0–4
M	Required Generation Number to Conjugate	15	5–20
K	Fitness Coefficient	0.2	0.1–1.0
P	Imbalance Coefficient	1	0.5–2
S	Probability of Conjugation	0.02	0.01–0.1

**Table 2:** Properties of individual ciliates during simulation.

Property	Abbreviation	Description
Generation Number	G	Number of times a given cell has divided since its most recent conjugation
Iteration Age	A	Number of iterations a given cell has passed through since its most recent conjugation
Chromosome Number	N	Number of unique chromosomes present in the MAC following sexual conjugation, remains constant until next conjugation
Copy Number	X	Number of copies of each chromosome present in the MAC following sexual conjugation, remains constant until next conjugation.
Elimination	E	Fraction of DNA eliminated at the most recent MIC to MAC transition
Fitness	F	Probability of division

The age of a cell since it originated from a conjugation event is measured with two different values: iteration age, **A**, as measured by number of iterations and generation number, **G**, as measured by the number of asexual divisions. The copy number, **X**, the chromosome number, **N**, and the elimination coefficient, **E**, are initialized for each member of the founding population respectively as **X_0_**, **N_0_**, and zero

This simulation was implemented in Python, and the source code is available upon request.

### Conjugation

Two parameters determine whether a cell conjugates at a given iteration, **M** and **S**, which are constant throughout the simulation. **M** is the required maturity, measured in generations, **G**, for a cell to undergo conjugation. That is, if **G** is less than **M**, than the cell cannot conjugate. If **G** is greater than or equal to **M**, then the probability that the cell conjugates in any given iteration is **S**.

During conjugation the copy number, **X**, and percent DNA eliminated, **E**, of the cell can ‘mutate’ randomly. Both new values are chosen from a normal distribution with mean equal to the parent value and standard deviation equal to the parameters **Sd_copy_** and **Sd_elim_**. These parameters are also held constant throughout the simulation. To preserve meaningful values for **E** and **X**, both are bounded below by zero. In addition, an **E** value greater than 0.98 is assumed lethal, thus 0.98 bounds **E** from above. If a new value chosen from the normal distribution lies outside of these bounds, the value is reassigned to the appropriate bound.

After conjugation, the iteration age and generation number of the cell are both reset to zero, and the number of chromosomes, **N**, is recalculated from **E** according to an exponential relationship (Eq. 2) and to account for the extra time taken to conjugate, the cell cannot divide until it has reached a certain iteration age, **I**.
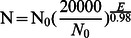
(2)


The equation forms an exponential relationship between the number of chromosomes and the amount of DNA eliminated, **E**. As **E** increases, so does the amount of fragmentation and thus chromosome number, **N**. There are two fixed points, which define the function completely: The first is at **E** = 0, where no DNA is eliminated, thus the chromosome number is equal to the initial number of chromosomes, **N**_0_. The second is at the point when all non-coding DNA has been eliminated; because some Spirotrich species eliminate up to 98% of the micronuclear DNA, we assume this point to be **E** = 0.98. The theoretical limit to the number of chromosomes is the number of genes, as no particular gene can be spread across multiple chromosomes. Thus at the maximum amount of DNA eliminated, the number of chromosomes is 20,000, approximately the number of genes in Spirotrichs.

Note that sexual conjugation in our simulation does not involve any exchange of information between cells. This avoids the complicated problem of selecting pairs of cells and averaging parameters.

### Division

All cells with iteration age greater than **I** have a chance to divide asexually if they have not conjugated this iteration. This probability is given by the fitness of the cell, **F**. When a cell divides, the iteration age and generation number increase by one. Both copy number, **X**, and the elimination parameter, **E**, do not change as these are defined when the MAC is generated from the MIC following conjugation. Note that by changing the generation number, the fitness of the cell is affected. An identical copy of the cell is then added to the population.

If a cell neither conjugates nor divides, then its iteration age – but not its generation number – increases by one; all of its other parameters remain unchanged. After each cell in the population is processed, 1,000 individuals are randomly selected to survive to the next generation, independent of their individual fitness values.

### Fitness

The fitness of each cell in the population is determined by two factors: the amount of DNA in the macronucleus, **F_DNA_**, and a measure of chromosome imbalance, **F_imb_**. The product of these two is used as the fitness, **F**. Perhaps the most straightforward way to measure these two factors is the brute force approach: to keep track of the copy number of each individual chromosome after asexual division. The copy numbers would vary according to a binomial distribution, as each individual chromosome would have a 50% chance of ending up in each daughter cell. However, there are several caveats to this brute force approach: First, as each cell can have thousands of distinct chromosomes type, monitoring the copy numbers becomes computationally expensive, to the point where simulations become impractical. Secondly, it is completely unknown how chromosomes fragment throughout evolution; thus the number of assumptions necessary may cast doubt on our results.

To preserve the simplicity and computational viability, we use population level measures to calculate the chromosome imbalances of macronucleus and the total amount of DNA in the macronucleus. We assume that the total amount of DNA in the macronucleus does not change during vegetative growth. While the number of a given chromosome can vary widely, the total is a sum of these variations, and thus will vary relatively little between conjugations. As these variations are lost in the regeneration of the macronucleus, these small variations will not accumulate, justifying our assumption. Thus, the fitness, **F_DNA_**, is measured as:
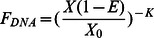
(3)where **X** is the copy number, **E** is the elimination coefficient, and **X_0_** is the initial copy number. The value of **K** defines how the DNA content influences fitness. This formula calculates the relative amount of macronuclear DNA in a given cell compared to that of a cell of the starting population. If a cell increases its total macronuclear DNA content, **F_DNA_** will decrease, representing the fitness costs of replicating large amounts of DNA. We assume that the founding population has a fitness of 1, and that its fitness cannot be improved by decreasing the DNA content of the MAC. Thus, while Equation 3 can adopt values greater than one, **F**_DNA_ is interpreted as a probability and bounded above by one.

As a proxy for the fitness costs of chromosome imbalances we used a modified version of Kimura's equation (Eq. 1). Although Kimura's equation gives the probability that a cell will not have lost a chromosome after a certain number of generations, we use it here as a measure of chromosome imbalances, as a growth in probability of chromosome loss should mirror the growth of chromosome imbalances via amitosis. Following conjugation, the cells start with a balanced MAC, and thus **F_imb_** = 1, as more and more amitotic divisions occur **F_imb_** decreases. The formula for **F_imb_** is thus:
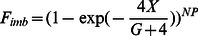
(4)where **X** is the copy number, **N** is the number of chromosomes, G is the generation number, and **P** is a parameter. The product of **F_imb_** and **F_DNA_** gives us the total fitness of a cell, **F**. Note that the specific contributions of **F_imb_** and **F_DNA_** to the total fitness can be weighted by the parameters K and P.

We run simulations with the given values (Table 1) as well as varying parameters individually. We repeated the simulation five times for each set of parameters. Figure 1 shows a flowchart of the simulations.

## Results

**Figure 2:**
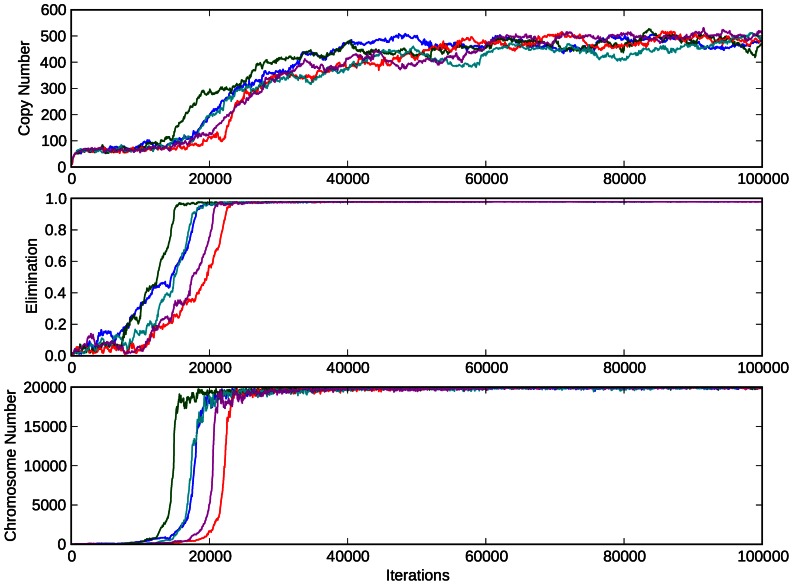
Simulation data for default values given in Table 1. The top, middle, and bottom graph show copy number, **X**, elimination coefficient, **E**, and chromosome number, **N**, respectively versus number of iterations. Each value represents the average of the population, and each colored line represents a different trial, all with the same parameters. Note that due to the stochastic nature of our simulation we see significant variation between trails using the same parameters, yet the final result is consistent.

For the default values (Table 1), the simulation followed the same pattern for each trial: Initially, the average value of **F_imb_** dropped and the copy number rose sharply, with the accompanying dip in **F_DNA_** From there, both the elimination coefficient, **E**, and the copy number, **X**, followed a sharp upward trend approaching an apparent stable distribution, with **E** remaining very close to its maximum value of 0.98, and **X** rising to approximately 500. We noted that the elimination coefficient tended to reach its stable distribution earlier than the copy number. Since the chromosome number, **N**, is a function of **E**, once the elimination coefficient reaches its stable distribution, tightly supported near 0.98, **N** quickly jumps to a stable distribution supported near 20,000 (Figure 2).

To assess the robustness of our simulation, ranges of values were tested for each parameter. We found that while different parameters affected the timing, duration, and variance of our results, in each case the same general patterns and final distribution were observed: high copy number, extensive elimination, and high number of different chromosome types.

## Discussion

Almost all ciliate macronuclei are able to divide; the single exception – Karyorelict ciliates – is thought to be the ciliate with the closest macronuclear organization to that of the ancestral ciliate [10]. In this species a new ‘nearly diploid’ macronucleus is generated from the micronucleus following each cell division.

In our model the initial copy number, **X_0_**, the chromosome number, **N_0_**, and elimination coefficient, **E**, are chosen to imitate a ‘nearly diploid’ [10] organization like that observed in Karyorelict ciliates. As these values approximate the likely nuclear organization prior to the MAC developing the ability to divide, they are the logical starting point of our simulation. Due to an intrinsic uncertainty with using modern organisms as a template for ancient ones, a range of values for **X_0_** and **N_0_** were also used. Note that very low values of X_0_ would result in population extinction as the imbalances inherent to amitosis lead to the loss of chromosomes after only a few divisions.

The default values of **M** and **I**, 15 generations before maturation and twice as long to conjugate than divide asexually, are consistent with existing literature and conservative [14]. The probability of conjugation, **S**, is difficult to track in a natural environment, and lab conditions cannot provide a reliable measure, thus we arbitrarily chose a 1/50 probability and tested a large range of values for this parameter.

The exact relationship between amount of DNA and fitness, as well as that between Kimura's equation and chromosome imbalance, represented as **K** and **P** respectively, is not known. Again, lacking concrete data, we chose arbitrary values and varied them in additional simulations. However, extremely small or high values were not used: given the stochastic nature of our simulation, small values would act as if there were no fitness dependence. Larger values would prevent significant changes in either elimination or DNA content in the time scale of our simulation. Note that the use of **K** and **P** also allows us to vary the respective weights of the two fitness functions.

Given the wide variation of copy number and DNA elimination among ciliate species and the consistency of these values within ciliate species, our model assumes that these features are genetically – or epigenetically – determined and can mutate. The rate of such mutations is unknown. The parameters **Sd_copy_** and **Sd_elim_** represent the standard deviations of the normal distribution used during mutation of copy number and elimination coefficient respectively. The values of these parameters were chosen as a trade-off between speed of convergence and clarity of data – large standard deviations lead to rapid convergence but to large fluctuations in the data, while small standard deviation values lead to very slow convergence – but this did not affect the final trends of large copy number, high fragmentation and numerous chromosome types.

In terms of the relationship between DNA elimination and chromosome fragmentation, an exponential function (Eq. 2) represents the best biological model as the behavior of this function approximately captures the important features of the available results. That is, for small amounts of DNA eliminated, there is only a small increase in the chromosome number, due to the effects of chromosome repair. When larger amounts of DNA are eliminated, a disproportionally larger increase in chromosome fragmentation occurs [26]. Note that this is a conservative assumption with respect to our model: an exponential relation may seem to support only a moderate level of elimination, as the costs of elimination increase quickly relative to the amount of DNA already eliminated. We assume that the elimination of 98% of the DNA in the MIC would lead to approximately an equal number of chromosomes and genes, ∼20000, and that an **E** of zero would lead to a MAC with the same number of chromosomes as the MIC, **N_0_.** These two fixed points are used to define the relationship between DNA elimination and chromosome fragmentation completely.

Varying the parameters of our simulation affects the variability and timing of our results, yet the final results are highly robust. In both the simulations and nature, Spirotrichs eliminate nearly all non-coding DNA in their MAC. Additionally, we observed a 50-fold increase in copy number during our simulation. While this generates only a fraction of the total ploidy observed in nature (∼500 versus thousands), the magnitude of this increase is still consistent with a clear evolutionary force in favor of high ploidy.

Our approach contains some limiting assumptions. In the simulation, a cell conjugates with itself, which is not an accurate representation of natural conjugation. Yet given the unknown nature of how attributes such as DNA elimination evolve, including a truly sexual process introduces more complexity, more necessary assumptions, and more parameters that are difficult to identify.

It is important to note that the model proposed here is only one of the ways by which a stable balance between copy number and chromosome fragmentation might have arisen. Other factors, like regulation of copy number during vegetative growth, could help explain the enormous variation in macronuclear organization in ciliates. In fact, regulatory mechanisms of copy number have been proposed for several ciliates with modest number of macronuclear chromosomes like *P. tetraurelia* [27] and *T. thermophila* [28], [29], [30]. Recently, Britto et al. proposed that selection against chromosome imbalances in dividing cells could help regulate DNA content at a population level in Tetrahymena [18]. Albeit these mechanisms seem to exist in the above ciliates, it is harder to fathom a mechanism capable to regulate the copy number of thousands of different chromosomes.

The unusual organization of macronuclei in some ciliates poses an interesting evolutionary question. As the extreme cases are present in divergent species, this suggests a selective advantage of this peculiar genomic organization [9]. Our model posits that the competing forces of chromosome imbalance and streamlining total DNA content in the MAC of ciliates gives rise to extreme polyploidy and chromosome fragmentation. Given the simplicity of our model and the consistency of our results across parameter space, we can conclude that this model presents a plausible description of the evolution of extremely fragmented macronuclei in ciliates.
